# Pain perception in personality disorders

**DOI:** 10.1371/journal.pone.0323004

**Published:** 2025-05-09

**Authors:** Spyridoula Bourtzoni, Phil Reed

**Affiliations:** 1 School of Psychology, Swansea University, Swansea, United Kingdom; 2 Department of Psychology, Swansea University, Swansea, United Kingdom; Nottingham Trent University School of Science and Technology, UNITED KINGDOM OF GREAT BRITAIN AND NORTHERN IRELAND

## Abstract

Research has focused on pain perception of individuals with Borderline Personality Disorder, but there is a lack of research regarding pain perception for other types of Personality Disorders. The present study explored associations between the perception of experimentally-induced acute pain of individuals without acute or chronic pain with Borderline, as well as Histrionic, and Schizotypal, Personality Disorders traits. The primary question of interest was whether any personality disorders were associated with altered pain perception. Fifty-two participants had pain induced by a cold-pressor task, and were evaluated for personality disorder traits. Psychophysiological perception of pain was measured using pain threshold and tolerance tests, subjective reports of pain were taken using the McGill Pain Questionnaire, and physiological aspects were measured using Galvanic Skin Response as an index of physiological arousal. The results showed significant associations between Histrionic Personality Disorder and subjective reports of the sensory aspects, and intensity, of pain, but not with psychophysiological or physiological responses (although caution is needed in interpreting the results of multiple tests). There were no significant associations regarding pain perception and Borderline, or Schizotypal, Personality Disorders. These results are preliminary, but provide novel suggestions regarding the impact of Personality Disorder on pain perception and guidance for future research has been provided.

## Introduction

Personality Disorders (PDs) are pervasive patterns of inner experiences and behaviours that significantly diverge from the expectations of a culture [1]. The estimated prevalence of PDs varies [2,3], but is generally as around 6% for both Cluster A (eccentric, unusual) and Cluster C (anxious and/or fearful), and 1.5% for Cluster B (dramatic, emotional, erratic). Some evidence suggests that individuals with PDs can experience greater subjective pain and/or exaggerate their pain scores [4–7]. Coupled with the facts that patients with PDs in general can be difficult to manage [8,9], and there has been a rise in opioid painkiller abuse [10], which is higher in some PDs [5], this makes managing their pain for those with PD further complicated [4].

The most studied PD with regard to pain perception is Borderline Personality Disorder (BPD), which reflects a disorder of mood and impulse control. The relationship between BPD and pain experience is somewhat unclear [7,11], although those with BPD traits do present with higher risk of prescription-opioid use disorders [5,9,12]. In a review of the area [7], greater levels of reported chronic headache were noted for those with BPD. Similarly, BPD patients reported higher pain scores and interference, compared to patients who screened negatively for BPD traits [5]. These BPD traits were associated with daily use of benzodiazepines and prescription-opioid dependence. However, no differences were observed in objective physical health and functioning between the BPD positive and negative groups. Individuals with BPD present reduced pain thresholds, relative to healthy controls, with the use of thermal grill illusion to induce both heat and cold pain [13]. However, on remission, similar levels of heat pain threshold were noted in BPD and healthy controls. Repetitive peripheral magnetic stimulation produces reports of less severe pain women with BPD, who presented higher pain thresholds than a control group [11].

In contrast to BPD, there are fewer studies regarding pain perception for individuals with Histrionic Personality Disorder (HPD), which involves an overwhelming desire to be noticed, often through displaying dramatic behaviour. Clinical observations suggest individuals diagnosed with, or having traits of, HPD report greater pain than other patients [4,6]. In combination with HPD’s psychopathology, these findings could lead to the assumption that individuals presenting traits of HPD may report greater pain than patients exhibiting other PDs. However, there have been few controlled studies of the relationship between reports of pain and levels of HPD personality traits (i.e., not just assuming a binary presence/absence of a disorder). Similarly, current literature is lacking concerning Schizotypal Personality Disorder (SPD) and pain perception. SPD involves displaying unusual beliefs and fear, as well as difficulty maintaining social relationships. However, as SPD shares similar psychopathologic characteristics with psychotic spectrum disorders, it might be suggested that individuals with SPD traits would report decreased pain intensity as several reviews have concluded that patients with schizophrenia experience and report reduced pain [14,15].

Thus, although pain perception for BPD has been investigated, there is little existing research on pain perception in patients with other PDs. To address this gap in the literature, during this study experimentally-induced acute pain was induced for individuals without acuate or chronic pain with the use of a cold-pressor task [16], which is a commonly employed technique to induce pain experimentally. The cold-pressor task involves immersion of a hand in cold water, and measuring the time until pain is reported (threshold) and until the hand is withdrawn (tolerance). The main objective was to explore the relationship between PD levels and pain as measured along multiple dimensions, reflecting the fact that pain is a multifaceted experience. These aspects involve not only psychophysiological reactions to stimuli, such as pain thresholds (the point at which a stimulus is reported as painful) and pain tolerance (the length of pain that can be withstood), but also physiological indices such a arousal (measured by Galvanic Skin Conductance), and subjective reactions as measured by standardized self-report psychometric tools.

Given these considerations, the current study explored the relationship between perception of experimentally-induced acute pain as measured by psychophysiological, physiological and subjective approaches, and three PDs Borderline (BPD), Histrionic (HPD), and Schizotypal (SPD), that have empirical and theoretical reasons to suggest they may be associated with altered pain reactivity and experience. In addition, a secondary objective was to assess any connection between personality measures and previous suicide attempts.

## Method

### Ethical considerations

The study protocol was reviewed and approved by the University, Department of Psychology, Ethics Committee (Ref: 0106). Participants were given full information regarding the study, the procedures, the inclusion- exclusion criteria, confidentiality and anonymity of their data, and their ability to withdraw from the study. Participants were asked if they completely understood the procedures involved in the study, and all the information provided to them, before giving written consent. Data collection commenced March, 2018, and completed in October, 2018.

### Participants

Fifty-two participants (31 female and 21 male) were recruited from a University campus using social media, advertisements, emails, and the participant pool website. Participants’ ages ranged from 18 to 31 years old, with a mean of 21 (SD=±2.43). G-Power calculations for 80% power, using a rejection criterion of *p* < .05, for a medium-large effect size (*f’* = .25, based on effect sizes noted previously for pain and personality [11,13]), suggests 48 participants are required for a multiple regression with three predictors. Given there are multiple PDs and outcomes involved, then it may be that an adjusted rejection rate could be considered, and a more conservative estimate may be given by taking the number of tests to be employed (8) and adjusting *p* appropriately (*p* = .05/8 = .006), which implies 74 participants would be needed. The only inclusion criterion of the study was that participants should be between 18 and 65 years old. Exclusion criteria included: a history of cardiovascular and/or neurological diseases, diabetes and frostbites, recent serious injuries, and ongoing or chronic pain. These were based on self-reports, and participants answering yes to questions about the presence of these conditions were excluded from analyses. No participants consumed alcohol, drugs or analgesic medications in the 12 hours before the experiment. No demographic data were obtained in terms of ethnicity and psychiatric history.

### Materials

**Demographic and background questions** were asked of the participants, including the age, gender, whether they had a history of cardiovascular and/or neurological diseases, frostbite, diabetes, recent serious injuries, psychological diagnoses, suicide attempts, and ongoing or chronic pain. All of these questions were answered as ‘yes’ or ‘no’.

**Personality Diagnostic Questionnaire-4** (PDQ-4 [17]) is a self-report questionnaire, based on DSM-IV Axis II PDs, and includes 99 true/false items, but only the questions relating to BPD (9 questions), HPD (8 questions), and SPD(9 questions), were employed for this study. Scores could range from 0 to 9, with a cut-off of 5 implying risk of the disorder.

**Short-Form McGill Pain Questionnaire** (SF-MPQ [18]) was used to assess pain experience of participants during the experiment. The SF-MPQ includes questions assessing three aspects of the subjective reports, through 15 descriptors of pain, each rated from 0 (none) to 3 (severe). Eleven descriptors represent the sensory dimension of pain experience (e.g., sharp, aching), and four assess the affective components of pain (e.g., fearful, punishing-cruel). A single 10 cm Visual Analogue Scale, ranging from “no pain” to “worst possible pain”, rates pain intensity.

**Cold**-Pressor **task** [16]: Τhe cold-pressor task involves two buckets of water, each measured to be 25 cm in diameter. One of the buckets contained room temperature water (18-20oC), and the other one contained water at a constant temperature of 2–4oC. A digital thermometer (Doutop Aquarium Thermometer) was used to ensure the steadiness of the water temperature throughout the experiment.

**Galvanic Skin Response** (GSR) was evaluated using the Shimmer3 GSR+ Unit device. Finger electrodes (MLT116F) were attached to the palmar surface of the intermediate phalanges of the first and third fingers of the non-dominant hand. Measurement of skin conductance was carried out by an AD Instruments PowerLab 2/25 data acquisition system (model ML825) and the accompanying skin conductance amplifier (ML116).

### Procedure

Participants were tested individually in a quiet and comfortable laboratory room. After consent, they were presented with the PDQ-4 questionnaire, in order to assess any BPD, HPD, and SPD traits. Upon completion of the questionnaire, the GSR device was set up. When the GSR device was set, the researcher asked participants to place their dominant hand in the bucket containing the room temperature water for one minute. The GSR levels obtained from that task were used to determine the baseline levels. During baseline determination, the researcher ensured that the temperature of the cold-water bucket was within the range of 2-4^o^C, by adjusting the temperature as needed (for example, by stirring or adding ice cubes). Following baseline, participants were asked to place their hand in the cold-water bucket. They were then instructed to state when their hand started to feel painful, and when they were about to remove it from the bucket. Once the cold-pressor task was completed, participants were asked to complete the SF-MPQ, based on their experience of the cold-pressor task. When all stages of the experiment had been completed, participants were debriefed.

### Statistical analyses

Data were entered and saved to SPSS (v26), which was used to perform all analyses. The variables were checked for missing data, and participants with more than 25% missing data were excluded. Skewness and kurtosis were calculated for all variables, and with one exception (threshold pain), were between no greater than -2 or + 2. Normality was tested using the Kolmogorov-Smirnov test, and in all cases but one (McGill sensory scale) did not deviate from a normal distribution. Correlations were tested using Pearson correlation coefficients, and regressions were used to determine if PD scores predicted the various aspects of pain. In all cases, the rejection criterion was *p* < .05, with appropriate Bonferroni corrections for multiple comparisons. These corrections were applied separately at the level of each analytic procedure using a different outcome variable rather than to all of the tests employed in the current analyses, as the latter may reduce power, and increase the chances of error.

## Results

The sample mean on the PDQ-4 for BPD was 2.55 (±1.86; range = 0–7), with 11/52 (21%) scoring above cut-off. The PDQ-4 sample mean for HPD was 2.44 (±1.36; range = 0–6), with 9/52 (17%) scoring above cut-off. The PDQ-4 sample mean for SPD was 2.67 (±1.66; range = 0–7), with 6/52 (11%) scoring above cut-off. Pearson correlations (using Bonferroni correction p = .05/3 = .016), revealed a significant positive associations between BPD and HPD (*r* = .357, *p* = .009), BPD and SPD (*r* = .433, *p* < .001), but not between HPD and SPD (*r* = .004, *p* > .30).

Table 1 displays the sample means (standard deviation) for measures of pain. Psychophysiological measures (threshold, tolerance) are reported in seconds. Physiological arousal was measured by GSR during cold-pressor task compared to room temperature (cold pressure GSR minus baseline GSR). Subjective aspects of pain (sensory, affective, intensity) were measured by the SF-MPQ. Table 1 also displays the correlations between these measures. Threshold for noticing pain did not correlate with any other measure. However, tolerance correlated positively with the physiological arousal and subjective intensity, but negatively with affective properties. Sensory experience correlated positively with affective properties, and affective experience correlated positively with reported intensity. Of the sample, 10/52 (19%) reported suicide attempts, which did not correlate with any measure of pain, using point biserial correlations.

**Table 1:** Sample means (standard deviation) for measures of pain. Psychophysiological measures (threshold, tolerance) are reported in seconds. Physiological arousal was GSR during cold-pressor task compared to room temperature. Subjective aspects (sensory, affective, intensity) were measured by SF-MPQ.

	Mean	(SD)	Tolerence	Arousal	Sen ory	Affective	Int sity	Suicide
Threshold (s)	30.02	(35.03)	-.016	-.125	-.043	-.249	-.074	-.089
Tolerance (s)	105.13	(109.96)		.276*	-.067	-.259	-.342**	_ .166
Arousal (GSR)	1.52	(5.78)			-.077	.021	.065	-.062
Sensory (SF-MPQ)	12.10	(5.22)				.338**	.227	-.188
Affective (SF-MPQ)	2.29	(2.04)					.344**	-.045
Intensity (SF-MPQ)	5.50	(2.11)						-.234

*p < .05; **p < .01; ***p < .001.

### Personality disorders and psychophysiological measures of pain (cold-pressor task)

To assess the degree that the PDs were associated with psychophysiological indices of pain two multiple regression were employed, both using BPD, HPD, and SPD as predictors, and one using threshold (time to withdraw hand during the cold-pressor task) as the outcome, and the other using tolerance (time to report pain during the cold-pressor task) as the outcome. As two outcomes were used a Bonferroni correction (*p* = .05/2 = .025 was applied). The model predicting pain threshold was not significant, *F* < 1, *R*^*2*^ = .032, with no individual predictors being significantly associated with pain threshold; BPD (*β*=-3.93, *p* = .228), HPD (*β*=2.57, *p* = .521), SPD (*β*=2.46, *p* = .467). Similarly, the model predicting tolerance was not significant, *F* < 1, *R*^*2*^ = .025, with no individual predictors being significantly associated with pain tolerance; BPD (*β*=-7.46, *p* = .466), HPD (*β*=-5.96, *p* = .635), SPD (*β*=4.02, *p* = .706).

### Personality disorders and physiological measures of pain (GSR)

Physiological arousal produced by pain was measured by the change in GSR during the cold-pressor task (mean = 12.29 ± 7.25) compared to the room temperature water (mean = 10.72 ± 5.22). This represented a significant increase in arousal during the cold pressure task, *t*(51)=1.99, *p* = .031, *d* = .26[*95%CI* = .014:.540]. However, a multiple regression using BPD, HPD, and SPD as predictors of GSR change was not significant, *F* < 1, *R*^*2*^ = .049, with no individual predictors being significantly associated with pain tolerance; BPD (*β*=-.42, *p* = .429), HPD (*β*=-.59, *p* = .369), SPD (*β*=.33, *p* = .559).

### Personality disorders and subjective measures of pain (SF-MPQ)

To assess the impact of PDs on subjective reports of pain, multiple regressions using BPD, HPD, and SPD as predictors were conducted for the SF_MPQ scores for sensory, affective, and intensity aspects of pain. As three outcomes were analysed a Bonferroni correction (*p* = .05/3 = .017) was applied. The model predicting the sensory aspects of pain approached significance, *F*(3,48)=2.44, *p* = .077, *R*^*2*^ = .131, with a significant association with HPD (*β*=1.38, *p* = .017), a marginal association with BPD (*β*=-.82, *p* = .078), but no association with SPD (*β*=.64, *p* = .183). The model predicting affective aspects of pain was not significant, *F* < 1, *R*^*2*^ = .006, and no individual predictors were associated with the affective aspects; BPD (*β* = .15, *p* = .939), HPD (*β*=.28, *p* = .906), SPD (*β*=.84, *p* = .675). The model relating PDs to subjective pain intensity approached significance, *F*(3,48)=2.25, *p* = .095, *R*^*2*^ = .123, with a significant association with HPD (*β*=.56, *p* = .016), but not BPD (*β*=-.22, *p* = .235), or SPD (*β*=.250, *p* = .201).

### Personality disorders and suicide attempts

A binominal logistic regression analysis investigated the relationship between the three PDs and whether the participants had made at least on suicide attempt in their past. The results of the analysis were statistically significant: *χ2*(3)=9.320, *p* = .025, *-2LL* = 41.59, with a significant association with BPD (*β*=.588, *p* = .027, *OR* = 1.80), but not HPD (*β*=.19, *p* = .550, *OR* = 1.20), or SPD (*β*=-.147, *p* = .590, *OR* = .863). The analysis explained 26.3% (Nagelkerke *R*^*2*^) of the variance in past suicide attempts, and correctly classified 81% of the cases.

## Discussion

The primary aim of the present study was to explore potential associations betwee perception of experimentally-induced pain and a range of personality disorders. The most important finding was a positive association between higher levels of histrionic personality disorder traits and subjective reports of sensory and intensity aspects of experimentally-induced pain (although caution should be applied when interpreting exploratory relationships based on multiple testing). No relationships were observed between participants’ subjective pain experience and the other personality disorders (BPD or SPD) studied. The association between HPD and the pain perception appears to corroborate several clinical case reports that there is a relationship between HPD and reports of pain experience [4,6].

Taking into account the psychopathology of HPD, it could be hypothesised that individuals with higher HPD traits could exaggerate their pain reports, perhaps in order to gain attention. This hypothesis could be strengthened by the results obtained from assessment of HPD characteristics and other pain perception, as HPD traits were found to be solely associated with the subjective reports. No other association between HPD traits and pain measurement was found, including the psychophysiological or physiological reactions to painful experience. The association between pain perception and HPD characteristics could be explained by possible biomedical or neuroanatomical differentiation of HPD individuals, compared to patients suffering from other forms of PDs. This hypothesis, though, needs to be tested in lager studies, in order to comprehend HPD’s neuroanatomical background, and to find any similarities or differences between HBP, BPD, and SPD.

Several studies have examined pain perception in patients with BPD and psychotic spectrum disorders. According to those studies, participants exhibiting BPD and SPD traits, should have presented a significant negative association between their PD traits and pain measurements ([8,11,13–15]. In the present experiment, no such indicators were observed. The present study, therefore, did not confirm previous literature concerning pain perception of individuals with BPD, or psychotic spectrum disorders. However, the results indicated that participants with BPD traits were more likely to have made a suicide attempt in the past, in contrast to HPD and SPD volunteers, a finding that is in accordance with BPD’s psychopathology.

Amongst the other findings of our study, there were positive correlations between BPD and HPD, and between BPD and SPD. Several studies have questioned the diagnosis of HPD, and have stressed the overlap between BPD and HPD psychopathology [19,20]. The relationship between BPD and SPD could be explained by the split of BPD, following the DSM-III diagnostic manual, into BPD and SPD. Data extracted from studies conducted in the past suggested that there might be an overlap between the two forms of PDs [21].

Our study presents with several limitations that could explain the conflicting results between our experiment and others found in the literature. Firstly, the study sample is considered to be small, and the sample presented an unequal sex ratio. Although adequately powered if the data had a large effect size, the current sample falls short for the medium effect size that was, in fact, obtained. This means that interpreting the null findings should be done with some caution. Test results might have been affected by the volunteers’ sex, as data from other studies have shown that male subjects demonstrate greater pain tolerance than females [22]. Additionally, the number of women taking oral contraceptives, nor the phase of the women menstrual cycle, were not measured, and other studies have shown that those factors could alter pain threshold and tolerance [23]. Another limitation of the study could concern the social status of the participants, which was not analysed, as it has been observed that those with lower socio-economic status have lowered tolerance to experimentally-induced pain [24]. The sampling method differed from some other experiments, as we did not have access in diagnosed BPD, HPD, and SPD patients, all of our participants were university students. Finally, it is worth mentioning that the participants’ psychiatric and prescription histories were not assessed. A positive psychiatric history or prescription medications could have, also, affected their responses or their pain perception.

Notwithstanding these considerations, given a reported increase of 70% in opioid-related drug misuse since 2015, and the prescription of over 23 million opioid-based painkillers in 2017 [10], these data emphasise the need for further investigation of the relationship between PDs and pain perception as these individuals may be at especially high risk of developing substance dependencies. The results of the study regarding the way individuals with HPD characteristics experience pain wait confirmation from larger studies, but suggest further work. Since current research data stress the fact that individuals with BPD and chronic pain conditions are more likely to develop prescription-opioid use dependence, more studies need to be conducted not only involving individuals with BPD traits, but also with SPD and HPD characteristics. Those studies could help the scientific community create guidelines regarding the pain assessment and management of individuals with such diagnoses.

### Consent for publication

All authors give consent for publication.
